# Vitamin K and muscle health: mechanisms and clinical perspectives in sarcopenia and beyond: narrative review

**DOI:** 10.3389/fnut.2026.1726483

**Published:** 2026-02-26

**Authors:** Xiaoyu Ran, Yuqi Jiang, Linxiu Mao, Xiuhua Chen, Dan Jing, Xun Tang, Jing Tan

**Affiliations:** 1 Clinical Medicine College, North Sichuan Medical College, Nanchong, Sichuan, China; 2 Department of Hematology, The Third People’s Hospital of Chengdu, Chengdu, Sichuan, China

**Keywords:** anti-inflammatory, antioxidant, muscle, sarcopenia, vitamin K, vitamin K–dependent proteins

## Abstract

Vitamin K, a fat-soluble micronutrient traditionally recognized for its role in blood coagulation, has increasingly been implicated as a micronutrient with emerging roles in skeletal muscle health. Experimental and clinical evidence now suggests that vitamin K influences skeletal muscle through both γ-carboxylation–dependent pathways—mediated by osteocalcin, matrix Gla protein (MGP), and growth arrest–specific 6 (Gas6)—and through non-carboxylation mechanisms, including anti-inflammatory, antioxidant, mitochondrial-regulatory, and ferroptosis-suppressing effects. Observational studies associate higher vitamin K intake and status with greater muscle strength, higher muscle mass, and better physical performance among older adults. However, findings from randomized controlled trials (RCTs) remain inconclusive, possibly due to differences in vitamin K isoforms, dosage, intervention duration, and study populations. Beyond age-related sarcopenia, vitamin K may also play a potentially protective role in muscle dysfunction associated with chronic diseases, including dialysis-related cramps and metabolic disorders. This review synthesizes recent mechanistic insights and clinical evidence, highlighting vitamin K as a biologically plausible contributor that is supported primarily by observational and mechanistic evidence for the prevention and management of sarcopenia and other muscle-related disorders, though its role remains incompletely validated.

## 1 Introduction

Sarcopenia, defined by the progressive decline of skeletal muscle mass, strength, and performance, represents a major contributor to frailty, disability, and mortality in aging populations (1–3). Its multifactorial pathogenesis involves inflammation, oxidative stress, and mitochondrial dysfunction, which together pose significant challenges for prevention and treatment (4–6). While exercise and protein intake remain the cornerstones of sarcopenia management, effective pharmacological or nutraceutical interventions are still lacking (7–9).

Vitamin K, long regarded primarily as a cofactor for the γ-carboxylation of coagulation factors (10), is increasingly recognized as an micronutrient in musculoskeletal health (11, 12). Mechanistic studies suggest that its biological effects are mediated primarily through carboxylation-dependent pathways (via osteocalcin, MGP, and Gas6) and secondarily through non-carboxylation mechanisms, including the regulation of inflammation, oxidative stress, mitochondrial homeostasis, and ferroptosis (10, 13).

Epidemiological studies support this notion, reporting that higher vitamin K status is associated with better physical performance and lower risk of mobility limitation in older adults (14, 15). However, randomized controlled trials evaluating vitamin K supplementation have produced inconsistent results, likely due to heterogeneity in study design, intervention type, and target populations (11, 12). Importantly, emerging data suggest that vitamin K may also modulate muscle outcomes in chronic disease states such as dialysis-related muscle dysfunction (16, 17). Furthermore, in clinical studies, the dosages of vitamin K1 have ranged from 80 μg to 1 mg per day, while those of menaquinone-7 (MK-7) have ranged from 61 μg to 375 μg daily; menaquinone-4 (MK-4) has been administered at doses significantly exceeding typical dietary intake, with no reported adverse effects (18).

Therefore, this review aims to systematically summarize the mechanistic insights and clinical advances of vitamin K in maintaining muscle health, focusing on its potential in sarcopenia and related myopathies.

The literature discussed in this narrative review was identified through searches of PubMed, Web of Science, and Scopus using combinations of keywords related to vitamin K, skeletal muscle, sarcopenia, muscle metabolism, inflammation, and mitochondrial function. The primary focus was on peer-reviewed articles published within the past 10–15 years, with earlier seminal studies included when necessary to provide historical context and mechanistic foundations. No predefined inclusion or exclusion criteria or quantitative synthesis was applied, consistent with the narrative and integrative nature of this review.

## 2 Vitamin K biology: types, distribution, and metabolism relevant to muscle health

### 2.1 Types and sources

Vitamin K comprises two primary natural isoforms: phylloquinone (vitamin K1) and menaquinones (vitamin K2, abbreviated as MK-n, where n represents the number of isoprene units). Among the 12 different types of MK (from MK-4 to MK-15), the most common types in the human body are short-chain MK-4 (which can be produced through the conversion of phylloquinone) and long-chain MKs, namely MK-7 to MK-10 (19, 20). Phylloquinone is abundant in green leafy vegetables such as spinach, kale, and broccoli, whereas menaquinones are synthesized by bacterial fermentation and found in foods such as natto, cheese, dairy, and meat products (13, 21). Among the menaquinones, MK-4 represents the major tissue form in mammals. It is biosynthesized from dietary K1 or menadione (K3) via the UbiA-prenyltransferase domain-containing protein 1 (UBIAD1) enzyme pathway (22–24). Importantly, MK-4 functions as an electron carrier within mitochondria, supporting ATP generation and reducing reactive oxygen species (ROS) (25). Compared with vitamin K1, vitamin K2—particularly the long-chain menaquinones such as MK-7—exhibits higher bioavailability and a longer plasma half-life, allowing it to accumulate preferentially in extrahepatic skeletal muscle and bone (21). These pharmacokinetic distinctions suggest that different vitamin K isoforms may exert tissue-specific effects, particularly in muscle metabolism and mitochondrial function.

### 2.2 Absorption, tissue distribution and metabolic conversion

Phylloquinone is absorbed in the jejunum and ileum, while menaquinone is absorbed from the distal intestine (26, 27). The distribution and transport of vitamin K are mediated by lipoproteins. Therefore, low-fat diets, fat-blocking supplements, antibiotic use, or bile acid sequestrants may hinder the absorption of vitamin K (28, 29). Vitamin K1 is preferentially retained in the liver, where it participates in the carboxylation of coagulation factors. In contrast, vitamin K2, after being absorbed in the intestine, can efficiently enter the bloodstream through enterocytes. This is due to the higher lipophilicity of long-chain menaquinones, which leads to their preferential transport via low-density lipoproteins (LDL) with a longer circulation time, thus enabling more effective redistribution from the liver to extrahepatic tissues compared to vitamin K1 (29, 30). Moreover, vitamin K1 is primarily associated with triglyceride-rich lipoproteins and is rapidly cleared by the liver (30). Collectively, these findings indicate that vitamin K is not limited to the regulation of coagulation functions in the liver. Its transformation and distribution in extrahepatic tissues, especially the efficient absorption and transport mechanisms of vitamin K2, may be of great significance to muscle function and energy balance.

### 2.3 Functional implications for muscle

All vitamin K isoforms serve as cofactors for γ-glutamyl carboxylase (GGCX), enabling activation of vitamin K–dependent proteins (VKDPs) such as osteocalcin, MGP, and Gas6, which have been implicated in muscle regeneration, vascular perfusion, and mitochondrial homeostasis (11, 13, 21). Beyond carboxylation, vitamin K also exerts anti-inflammatory, antioxidant, and ferroptosis-suppressing effects in preclinical studies, thereby potentially supporting muscle mass and function in aging and disease (31–33).

## 3 Mechanistic insights: vitamin K in muscle health

Vitamin K may influence skeletal muscle through two complementary mechanisms: carboxylation-dependent pathways, which regulate vitamin K–dependent proteins (VKDPs), and carboxylation-independent pathways, which modulate inflammation, oxidative stress, ferroptosis, mitochondrial function, metabolism, and calcium balance. These effects intersect with the major biological processes implicated in sarcopenia.

### 3.1 Carboxylation-dependent pathways

**FIGURE 1:**
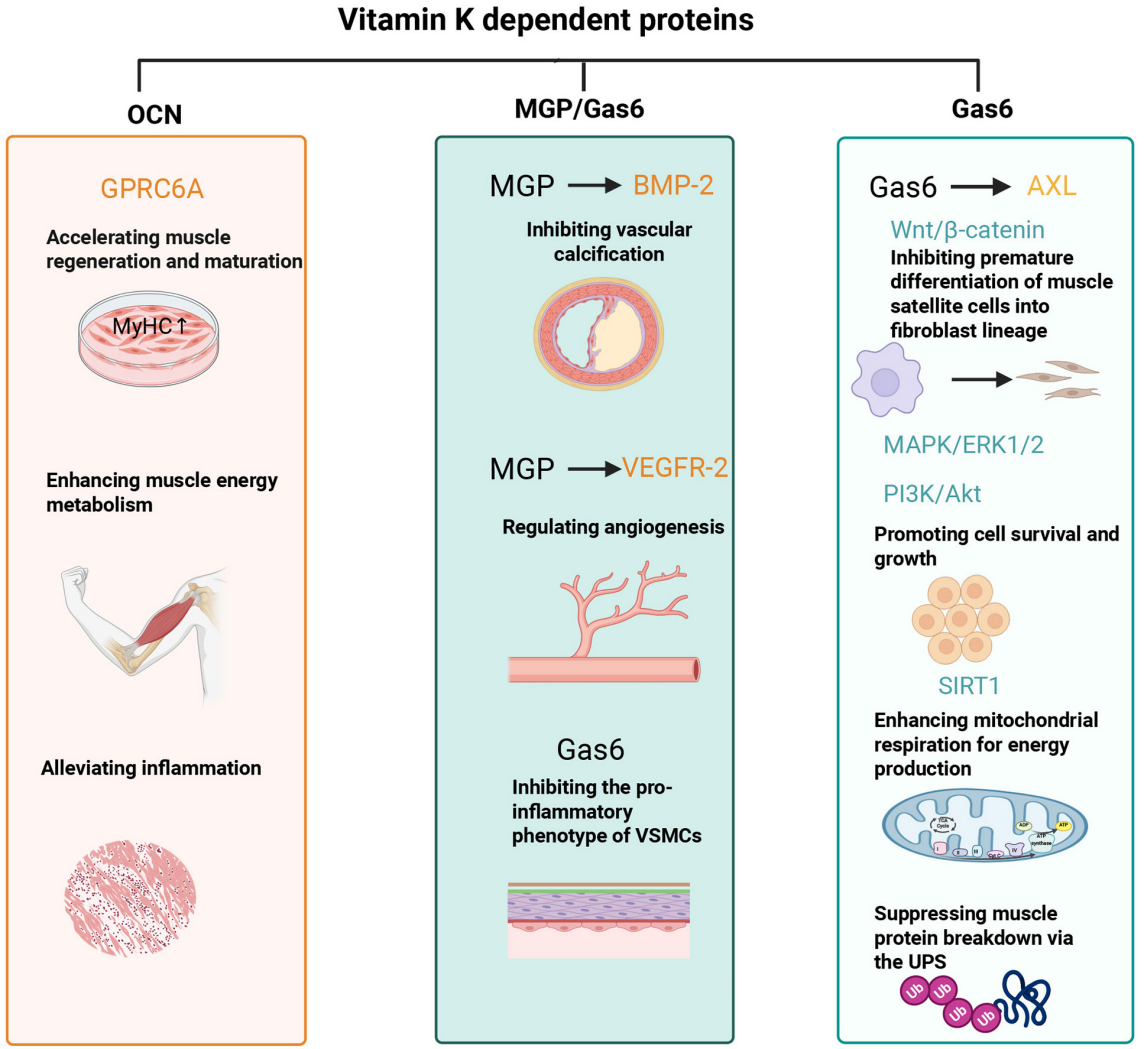
Vitamin K affects muscle health through vitamin K-dependent carboxylated proteins. OCN, osteocalcin; GPRC6A, G protein-coupled receptor class C group 6 member A; MyHC, myosin heavy chain; MGP, matrix Gla protein; BMP-2, bone morphogenetic protein 2; VEGFR-2, vascular endothelial growth factor receptor 2; Gas6, growth arrest–specific 6; SIRT1, sirtuin 1; Wnt/β-catenin, Wnt/beta-catenin signaling pathway; MAPK/ERK1/2, mitogen-activated protein kinase/extracellular signal-regulated kinase 1/2; PI3K/Akt, phosphatidylinositol 3-kinase/protein kinase B; VSMCs, vascular smooth muscle cells; UPS, ubiquitin-proteasome system. This figure was created with BioRender.com.

Vitamin K is a cofactor for γ-glutamyl carboxylase, which activates VKDPs. Several VKDPs play pivotal roles have been implicated in experimental models in muscle regeneration, vascular health, and mitochondrial activation process of these VKDPs and their functional relevance to muscle health are visualized in Figure 1.

#### 3.1.1 Osteocalcin

Osteocalcin, a key VKDP secreted by osteoblasts, functions as a hormone and exerts significant regulatory effects during muscle regeneration and functional recovery (34). Research utilizing a muscle injury model in aged mice has revealed that osteocalcin is associated with enhanced muscle fiber regeneration, reduced fibrosis in injured muscle tissue, and improved muscle contraction strength and endurance (34). Mechanistically, osteocalcin activates the G protein-coupled receptor class C group 6 member A (GPRC6A) receptor on muscle cell membranes, which in turn promotes the proliferation and differentiation of muscle stem cells and increases the expression of myogenesis-related genes [such as myosin heavy chain (MyHC)]. This process may facilitate muscle fiber regeneration and maturation (34). Additionally, osteocalcin has been reported to attenuate the inflammatory response after muscle injury by reducing inflammatory cell infiltration, thereby improving the inflammatory microenvironment of muscle tissue and further decreasing fibrosis (34).

Beyond its potential role in muscle regeneration, osteocalcin functions as a key regulator of intracellular energy homeostasis. It has been shown to enhance glucose uptake by muscle cells, improve insulin sensitivity, and modulate energy metabolism pathways. By boosting mitochondrial activity and increasing energy production, osteocalcin may contribute to energy expenditure, ultimately supporting muscle endurance and exercise capacity (35, 36). Furthermore, research using the Ocn−/− mouse model has found that osteocalcin deficiency leads to impaired glucose uptake in skeletal muscle, a phenotype that can be reversed by exogenous osteocalcin supplementation (35). Collectively, these findings suggest that this skeletal-derived hormone-mediated bone-muscle interaction may contribute to the maintenance of the quality and function of adult and aged skeletal muscle, and its deficiency may predispose to age-related muscle loss (37). However, direct demonstration of these effects in human skeletal muscle is limited.

#### 3.1.2 Matrix Gla protein (MGP)

MGP may influence muscle health indirectly by preventing vascular calcification and maintaining blood flow. Elevated dephospho-uncarboxylated MGP (dp-ucMGP), reflecting poor vitamin K status, is associated with lower skeletal muscle mass index and reduced physical performance (38). MGP inactivation has been linked in experimental models to peripheral artery disease (PAD), leading to ischemic muscle injury characterized by increased apoptosis, fibrosis, iron deposition, and impaired satellite cell differentiation (39).

Mechanistically, MGP inhibits vascular calcification by binding to bone morphogenetic protein-2 (BMP-2), blocking pathological calcium deposition (40–43). MGP also regulates angiogenesis by controlling the expression of Vascular Endothelial Growth Factor A (VEGF-A) and its receptor vascular endothelial growth factor receptor 2 (VEGFR2), thereby maintaining normal vascular formation (44, 45). Defective activation of MGP due to vitamin K deficiency may contribute to peripheral vascular disease and tissue ischemia, thereby accelerating muscle atrophy and sarcopenia (38). Thus, patients undergoing long-term treatment with vitamin K antagonists (e.g., warfarin) face an increased risk of systemic vascular calcification due to the inhibition of MGP (46), and may plausibly experience downstream muscle functional consequences, although direct causal evidence in humans is limited.

#### 3.1.3 Growth arrest–specific protein 6 (Gas6)

Gas6, a VKDP secreted by vascular smooth muscle cells, signals via family receptors TAM (Tyro3, Axl, Mer) with Axl, being prominently expressed in skeletal muscle (47, 48). Activation of the Gas6/Axl signaling axis has been shown in experimental systems to suppress pro-inflammatory pathways, preserve mitochondrial function, and reduce oxidative stress—key for muscle cell homeostasis (49–51).

In skeletal muscle, Gas6/Axl signaling stimulates the mitogen-activated protein kinase (MAPK)/extracellular signal-regulated kinase 1/2 (ERK1/2) pathway (involved in proliferation) and the phosphatidylinositol 3-kinase (PI3K)/protein kinase B (Akt) pathway (involved in survival) cascades in preclinical models, promoting cell proliferation and survival (49, 50). Additionally, by activating the Gas6/Axl signaling axis, Gas6 inhibits the activity of the Wnt/β-catenin signaling pathway, thereby preventing the premature differentiation of muscle satellite cells into fibroblast lineages and consequently avoiding the depletion of the satellite cell pool and the reduction of muscle regeneration capacity (48, 52–54). Furthermore, sirtuin 1 (SIRT1), a downstream component of Gas6/Axl signaling, enhances mitochondrial respiration for energy production and suppresses muscle protein breakdown via the ubiquitin-proteasome system (47, 55). While these pathways provide biological plausibility, their contribution to human sarcopenia remains largely inferential and requires further validation.

### 3.2 Carboxylation-independent pathways

Beyond its enzymatic role in VKDP activation, vitamin K has been reported to exerts pleiotropic actions through non-carboxylation mechanisms in preclinical studies, influencing inflammation, oxidative stress, ferroptosis, metabolism, and mitochondrial function.

#### 3.2.1 Anti-inflammatory effects

Chronic inflammation is a key mechanism underlying the development and progression of sarcopenia (56–58). Inflammatory stimulation alters the microenvironment of skeletal muscle cells: pro-inflammatory cytokines activate signaling pathways such as nuclear factor-κB (NF)-κB, Janus kinase/signal transducer and activator of transcription (JAK/STAT), p38 mitogen-activated protein kinase (p38MAPK), and the ubiquitin-proteasome system (UPS). These activations disrupt the balance between protein synthesis and degradation in skeletal muscle, enhance proteolysis, and inhibit protein synthesis, ultimately leading to skeletal muscle atrophy (56, 59, 60).

Vitamin K (particularly MK-4) has been reported to inhibit nuclear factor κB (NF-κB) activation by blocking inhibitor of κB kinase α/β (IKKα/β) phosphorylation, thereby suppressing interleukin-6 (IL-6), tumor necrosis factor-α (TNF-α), and interleukin-1β (IL-1β) production (13, 61, 62). Further studies indicate that vitamin K2 also enhances its anti-inflammatory effects by inhibiting MAPK/NF-κB pathway activation triggered by downregulation of intracellular GPX4 expression (63). Consistent with these mechanisms, observational studies indicate that higher vitamin K levels are significantly associated with reduced systemic inflammation (64), animal studies further confirm that vitamin K supplementation alleviates LPS-induced muscle injury by reducing cytokine release and restoring muscle mass (31, 65). Therefore, vitamin K may attenuate chronic inflammation-associated dysregulation of skeletal muscle protein metabolism through multi-targeted inhibition of key inflammatory signaling pathways such as NF-κB and reduction of pro-inflammatory cytokine release, suggesting its potential role in the prevention and management of sarcopenia.

#### 3.2.2 Antioxidant and ferroptosis inhibition

Ferroptosis, an iron-dependent form of cell death marked by lipid peroxidation and ROS accumulation, has been implicated in the pathogenesis of multiple muscle-related disorders, including sarcopenia, cardiomyopathy, and amyotrophic lateral sclerosis (ALS) (66, 67). Additionally, ferroptosis increasingly is recognized as a potential contributor to age-related muscle loss (68, 69).

**FIGURE 2:**
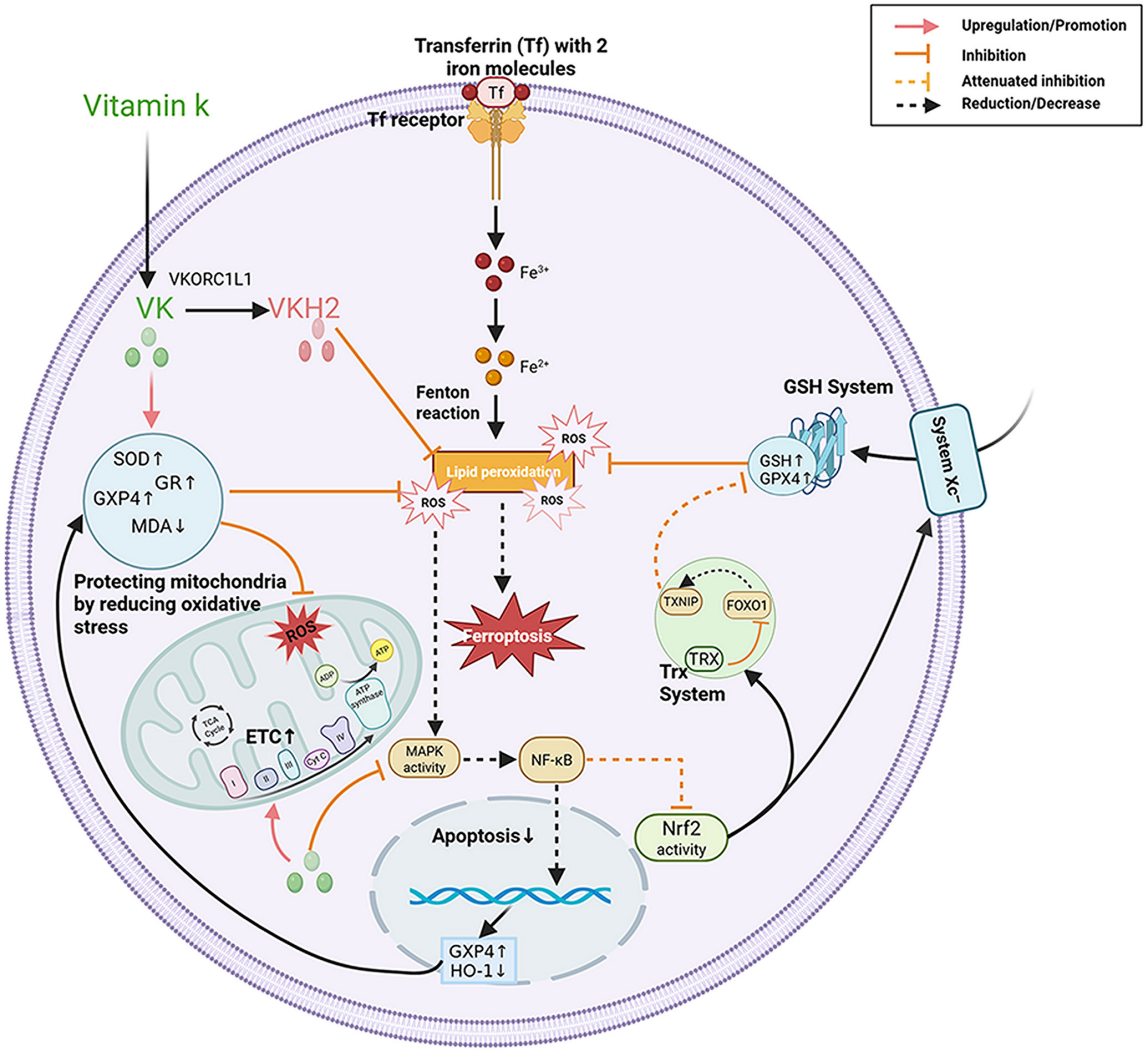
Vitamin K exerts pleiotropic effects through direct antioxidant, anti-inflammatory, ferroptosis-suppressing on muscle cells. Red solid lines indicate upregulation/promotion; yellow solid lines with a terminal bar indicate inhibition; yellow dashed lines with a terminal bar indicate attenuation of inhibition, and black dashed lines indicate reduction/decrease. VK, vitamin K; VKORC1L1, vitamin K epoxide reductase complex subunit 1 like 1; VKH_2_, vitamin K hydroquinone; SOD, superoxide dismutase; GR, glutathione reductase; GPX4, glutathione peroxidase 4; GSH, glutathione; MDA, malondialdehyde; ROS, reactive oxygen species; MAPK, mitogen-activated protein kinase; NF-κB, nuclear factor kappa B; Nrf2, nuclear factor erythroid 2-related factor 2; HO-1, heme oxygenase-1; TRX, thioredoxin; TXNIP, thioredoxin-interacting protein; FOXO1, forkhead box O1; ETC, electron transport chain. This figure was created with BioRender.com.

Vitamin K1 has been reported to attenuate ferroptosis-related oxidative injury in experimental systems by enhancing antioxidant enzymes superoxide dismutase (SOD), glutathione reductase (GR), while reducing the lipid peroxidation product malondialdehyde (MDA) (31). Similarly, vitamin K2 (MK-4) has been shown in preclinical models to reduce ferroptosis markers by upregulating glutathione peroxidase 4 (GPX4), a key enzyme that prevents lipid peroxidation and ferroptotic cell death, while also decreasing iron levels and the oxidative stress markers MDA (70, 71). The active reduced form of vitamin K is vitamin K dihydroquinone (VKH_2_), which is produced by the action of vitamin K epoxide reductase complex subunit 1-like 1 (VKORC1L1) and exhibits measurable antioxidant capacity independent of the glutathione (GSH)/GPX4 pathway. By directly scavenging ROS and maintaining iron metabolic balance, vitamin K hydroquinone may inhibit ferroptosis and protect muscle tissue from oxidative stress-induced damage (71, 72). Vitamin K has also been shown protects mitochondria by reducing ROS, maintaining membrane potential, and preventing fragmentation in preclinical studies, thereby potentially improving energy metabolism and reducing ferroptotic damage (25, 73, 74). Taken together, the antioxidant effect of vitamin K is one of the core mechanisms underlying its inhibition of ferroptosis—acting through two complementary pathways: it directly scavenges reactive oxygen species (ROS) and maintains iron metabolic balance via its active form vitamin K dihydroquinone (VKH_2_), while also upregulating the activity of antioxidant enzymes and reducing lipid peroxidation to further decrease ROS production. These dual pathways suppress oxidative stress and ultimately attenuate the ferroptosis process. These multiple regulatory mechanisms of vitamin K in ferroptosis -related pathways are summarized in Figure 2. Importantly, the majority of this evidence is derived from cell culture and animal studies, and direct validation in human skeletal muscle tissue remains lacking.

In addition to direct antioxidant effects, vitamin K influences cellular signaling pathways associated with ferroptosis. For instance, on the one hand, vitamin K can reduce the production of ROS induced by ferroptosis in experimental systems, thereby inhibiting the activation of the MAPK cascade by ROS at the source (an indirect mechanism) (75); on the other hand, preliminary studies suggest that it may exert direct regulatory effects—for instance, vitamin K2 inhibits the phosphorylation activity of key kinases in the MAPK pathway (e.g., p38) (70), and excessive activation of MAPK further drives the nuclear translocation of NF-κB (a pathway that promotes apoptosis and muscle damage) (76–80). Meanwhile, vitamin K2 can directly target IKKα/β, an upstream regulator of NF-κB, inhibiting its phosphorylation to block the degradation of Inhibitor of nuclear factor kappa-B alpha9 (IκBα), thereby reducing the activation and transcriptional function of NF-κB (61, 81). On this basis, vitamin K2 may upregulate the transcriptional expression of glutathione peroxidase 4 (GPX4) and downregulate the protein level of the pro-oxidant protein heme oxygenase-1 (HO-1) by reducing NF-κB activation, further mitigating iron-mediated oxidative stress (82, 83). This dual inhibitory effect—“direct regulation of key MAPK/NF-κB molecules + indirect blocking of ROS signaling”—subsequently relieves the inhibition of the nuclear factor erythroid 2-related factor 2 (Nrf2) pathway and promotes its activation (63, 70, 84, 85).

Furthermore, as a transcription factor, Nrf2, which not only regulates the glutathione antioxidant system but also modulates the thioredoxin antioxidant system, plays a crucial role in cellular defense against exogenous toxins and oxidative stress (85). The thioredoxin antioxidant system, through its thioredoxin (Trx)–mediated antioxidant function, directly inhibits the activity of forkhead box O1 (FOXO1), thereby reducing its transcriptional activation of the thioredoxin-interacting protein (TXNIP) gene. This cascade ultimately decreases TXNIP expression levels and diminishes TXNIP’s inhibitory effect on glutathione metabolism, thereby potentially ameliorating ferroptosis in skeletal muscle satellite cells (86). Together, these preclinical mechanisms suggest that vitamin K may function as a modulator of ferroptosis, mitigating oxidative damage and preserving muscle integrity, and signaling interactions are also summarized in Figure 2. However, whether such pathways are sufficiently engaged *in vivo* in human skeletal muscle to influence clinical sarcopenia outcomes remains uncertain.

#### 3.2.3 Metabolic regulation and muscle growth

**FIGURE 3:**
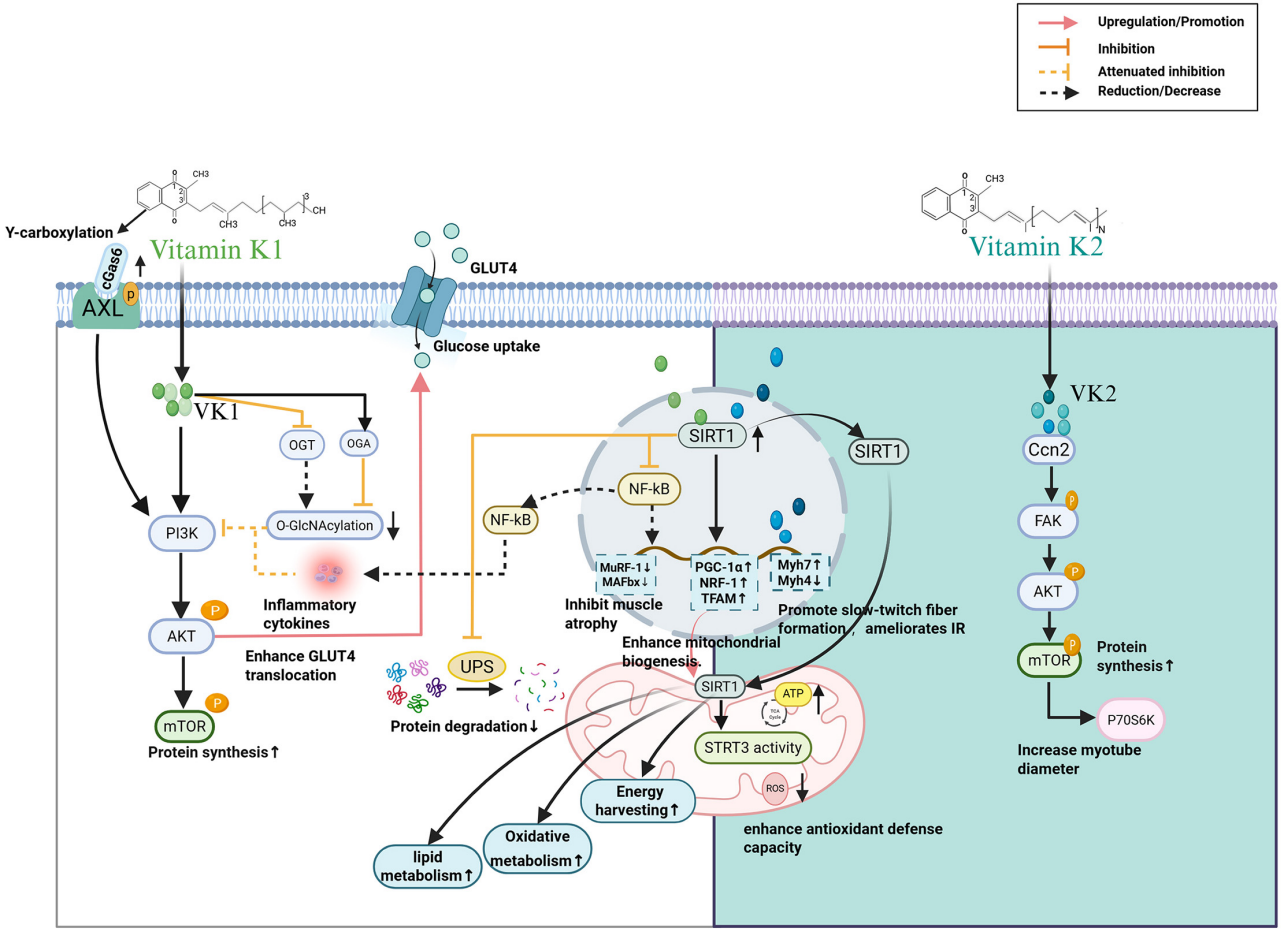
Vitamin K promotes muscle growth and regulates muscle cell metabolic function (see Figure 2). VK1, vitamin K1; VK2, vitamin K2; AXL, AXL receptor tyrosine; cGas6, carboxylated growth arrest–specific 6; PI3K, phosphatidylinositol 3-kinase; AKT, protein kinase B; mTOR, mammalian target of rapamycin; OGT, O-GlcNAc transferase; OGA, O-GlcNAcase; GLUT4, glucose transporter 4; SIRT1, silent information regulator 1; SIRT3, silent information regulator 3; NF-κB, nuclear factor kappa B; SOD2, superoxide dismutase 2; ROS, reactive oxygen species; Ccn2, connective tissue growth factor; FAK, focal adhesion kinase; P70S6K, p70 ribosomal S6 kinase; MuRF-1, muscle RING-finger protein 1; MAFbx, muscle atrophy F-box protein; PGC-1α, peroxisome proliferator-activated receptor-γ coactivator-1α; NRF-1, nuclear respiratory factor-1; TFAM, mitochondrial transcription factor A; MyH7, myosin heavy chain 7; MyH4, myosin heavy chain 4; IR, insulin resistance. This figure was created with BioRender.com.

Vitamin K has been proposed to influence muscle metabolism and anabolic signaling in experimental studies. Emerging evidence highlights its significance in modulating key signaling pathways, improving glucose metabolism, reducing muscle atrophy, and enhancing mitochondrial function. The specific roles of vitamin K in these processes and their associated molecular targets are presented in Figure 3.

Vitamin K1 has been shown in preclinical models to activate the PI3K/Akt/mTOR and SIRT1 signaling pathways, promoting protein synthesis, glucose uptake, and mitochondrial biogenesis (31, 65), vitamin K1 also regulates muscle protein degradation by downregulating atrophic markers such as muscle RING-finger protein-1 (MuRF-1) and muscle atrophy F-box (MAFbx, also known as atrogin-1) via the SIRT1/NF-κB pathway—both of which are significantly elevated during muscle atrophy (31, 87, 88). SIRT1, a deacetylase involved in the regulation of inflammatory responses, apoptosis, and energy metabolism, can antagonize NF-κB-induced inflammatory responses, with NF-κB being a key switch in the inflammatory response (89, 90). Furthermore, the activation of the SIRT1 signaling pathways by vitamin K1 not only reduces the expression of inflammatory cytokines associated with this pathway but also overcomes the inhibition of the PI3K/Akt/mTOR pathway by inflammatory cytokines, thereby helping to counteract the inhibitory effect of inflammation on muscle protein synthesis (31, 65, 91). Moreover, SIRT1 can also inhibit the ubiquitin-proteasome system, reducing muscle protein degradation. This dual regulation of synthesis and degradation is crucial for maintaining muscle mass, especially in catabolic states such as cachexia (13, 73). Nonetheless, these findings should be interpreted as mechanistic plausibility rather than direct evidence of clinical efficacy.

In models of insulin resistance, vitamin K1 enhances glucose transporter type 4 (GLUT4) translocation through carboxylated Gas6/Axl/PI3K/Akt signaling, improving glucose metabolism and muscle anabolism (92). Additionally, vitamin K may reduce protein O-GlcNAcylation by inhibiting O-GlcNAc transferase (OGT) and activating O-GlcNAcase (OGA); this reduction in modification levels relieves the inhibitory effect of O-GlcNAcylation on the PI3K/Akt/GLUT4 pathway, thereby restoring the expression and function of PI3K, Akt, and GLUT4, and enhancing the pathway’s glucose uptake capacity (92). Collectively, these metabolic effects suggest that vitamin K supports anabolic processes and suppresses muscle breakdown, linking it to a potential mechanistic link with sarcopenia-related metabolic dysregulation.

Zhang et al. (54) found that vitamin K2 could increase skeletal muscle mass, reduce fat accumulation, and also lower glucolipid metabolism parameters, including blood glucose (*p* < 0.01), glycated hemoglobin (HbA1c), the homeostasis model assessment of insulin resistance (HOMA-IR, *p* < 0.01), and serum lipid levels. Vitamin K2 targets through cellular communication network factor 2 (Ccn2), promoting the phosphorylation of proteins in the focal adhesion kinase (FAK)–Akt–mTOR–p70 ribosomal S6 kinase (p70S6K) pathway, which enhances protein synthesis, increases myotube diameter, and enlarges skeletal muscle fibers. This plays a crucial role in maintaining the balance between protein synthesis and degradation in skeletal muscle (54, 93, 94). Furthermore, vitamin K2 has been shown to upregulates slow-twitch muscle fiber genes (such as Myh7) while downregulating fast-twitch muscle fiber genes (such as Myh4) (73).

Mitochondrial dysfunction is a significant contributor to reduced muscle mass and contractility. Both vitamin K1 and vitamin K2 have been shown to play important roles in mitochondrial health in preclinical studies by upregulating SIRT1 protein expression, improving mitochondrial oxidative metabolism, and promoting the formation of slow-twitch (type I) muscle fibers, which are associated with enhanced oxidative capacity and endurance (11, 73, 93). SIRT1 activation enhances mitochondrial biogenesis by upregulating genes such as peroxisome proliferator-activated receptor-γ coactivator-1α (PGC-1α), nuclear respiratory factor-1 (NRF-1), and mitochondrial transcription factor A (TFAM), while also reducing ROS through antioxidant defenses, enhancing fat metabolism, and regulating mitochondrial respiration to optimize energy harvesting (55, 95). Vitamin K2 functions as an electron carrier in mitochondria, improving the efficiency of ATP production and reducing ROS accumulation. It also improves mitochondrial dysfunction and mitochondrial antioxidant defenses by activating the SIRT1/SIRT3 signaling pathway (73).

### 3.3 Calcium homeostasis regulation

Calcium ions (Ca^2+^) play pivotal roles in skeletal muscle, and maintaining their homeostasis is essential for normal muscle contraction, protein synthesis and degradation, fiber type transformation, and mitochondrial function (96). Dysregulation of calcium homeostasis is a common pathophysiological feature of multiple muscle diseases, including hypotonia, myopathy, muscular dystrophy, cachexia, and sarcopenia (97). Excessive intracellular calcium accumulation can result in mitochondrial calcium overload, leading to increasing ROS production, damaging mitochondrial membranes, inhibiting ATP synthesis, and ultimately causing cellular energy metabolism disorders—weakening muscle endurance and strength (97).

Clinical evidence supports a potential role for vitamin K2 (MK-7) in regulating intracellular calcium homeostasis in skeletal muscle. A randomized clinical trial showed that vitamin K2 supplementation significantly reduced the frequency, intensity, and duration of nocturnal leg cramps in elderly populations (98). Another double-blind trial found that MK-7 supplementation decreased the frequency and severity of muscle cramps in hemodialysis patients (16).

Animal studies on vitamin K3 intervention revealed it reduces intracellular calcium concentrations and weakens smooth muscle contraction by interfering with calcium transport—specifically inhibiting calcium influx and sarcoplasmic reticulum calcium release (99, 100). This provides additional evidence for vitamin K’s role in regulating calcium homeostasis and muscle function.

Notably, vitamin K-dependent processes may also impact muscle calcium regulation indirectly. In insulin-resistant mouse models, VKDC regulates β-cell Ca^2+^ flux, with γ-carboxylated ERGP (a vitamin K-dependent protein) suppressing store-operated calcium entry (SOCE) by reducing stromal interaction molecule 1 (STIM1)/Orai1 puncta formation (88, 101).

## 4 Vitamin K in muscle-related diseases

### 4.1 Sarcopenia

Sarcopenia, an age-related syndrome marked by the progressive decline in skeletal muscle mass, strength, and function, substantially impairs the quality of life in older adults. It contributes to frailty, mobility impairments, falls, fractures, and increased mortality, while also elevating healthcare costs (1–3). The underlying mechanisms of sarcopenia include genomic instability, telomere attrition, epigenetic dysregulation, and cellular senescence (6). Importantly, sarcopenia is not a single uniform outcome but a multidimensional clinical entity encompassing muscle quantity (mass), muscle strength, physical performance, and patient-relevant functional symptoms, and evidence for vitamin K may differ substantially across these domains. Accumulating evidence links low vitamin K status to increased muscle loss and functional decline, highlighting its potential role in combating sarcopenia (14, 102–104). Higher dietary vitamin K intake (70–100 μg/day) is associated with greater lean mass, improved strength, and reduced fall risk (15, 105). However, observational studies have most consistently reported associations with physical performance measures, grip strength, and fall-related outcomes (15, 102, 104), whereas associations with muscle mass have been more variable depending on assessment method, population characteristics, and adjustment for confounding factors. Therefore, observational evidence suggests that vitamin K status may be more strongly linked to functional performance and frailty-related outcomes than to muscle quantity alone.

In light of the mechanistic evidence discussed earlier, vitamin K may demonstrate the potential to mitigate sarcopenia through its antioxidant, anti-inflammatory, ferroptosis-modulating, and mitochondrial-supportive effects. These mechanisms provide biological plausibility for a role of vitamin K in sarcopenia-related pathways. However, mechanistic plausibility should not be interpreted as proof of uniform clinical benefit across all sarcopenia components. Interventional evidence remains limited. To date, randomized controlled trials have more frequently demonstrated potential benefits in selected symptom-based outcomes, particularly muscle cramps and related functional discomfort, while effects on sarcopenia defining endpoints such as muscle mass, strength, and physical performance have been inconsistent. Future research should, on one hand, explore the molecular mechanisms underlying its actions and validate its clinical applications in sarcopenia management. on the other hand, clinical trials should clearly distinguish primary endpoints across sarcopenia domains (mass, strength, performance, falls, and symptom-based outcomes), apply standardized diagnostic criteria, and stratify participants by baseline vitamin K status to determine which components of sarcopenia are most responsive to vitamin K interventions.

### 4.2 X-linked myotubular myopathy (XLMTM)

X-linked myotubular myopathy (XLMTM) is caused by mutations in the MTM1 gene, which encodes myotubularin—a phosphatase that dephosphorylates phosphoinositides, particularly phosphatidylinositol 3-phosphate (PI3P) and phosphatidylinositol 3,5-bisphosphate (PI3,5P2) (106, 107). These mutations lead to uncontrolled activity of vacuolar protein sorting 34 (VPS34) kinase, resulting in excessive PI(3)P production that hinders normal muscle development (108).

Notably, a synthetic vitamin K derivative, menadione sodium bisulfite (MSB), has been shown to inhibit VPS34 kinase—a key regulator of PI(3)P accumulation in XLMTM (108). By inhibiting and inactivating VPS34, MSB reduces PI(3)P levels, allowing muscles to resume normal growth (108). In an XLMTM animal model (Mtm1 knockout mice), MSB administration via drinking water significantly prolonged survival, increased body weight, enhanced muscle strength, and improved muscle histology (larger fibers, fewer abnormal mitochondria, corrected nuclear positioning) (108). These preclinical findings suggest a potential therapeutic avenue for XLMTM, though further experimental and clinical studies are needed to verify MSB’s potential and safety.

## 5 Clinical studies and nutritional interventions

### 5.1 Observational studies

Multiple clinical observational studies have demonstrated a significant association between vitamin K levels and physical function. A study involving 1,089 community-dwelling older adults (mean age 74 years) found that higher plasma vitamin K1 concentrations were significantly associated with better scores on the Short Physical Performance Battery (SPPB), suggesting that vitamin K1 may be positively correlated with maintaining physical function in older adults (104).

Research by Sim et al. (15) further confirmed the link between dietary vitamin K intake, physical function, and fall risk in older women. In a long-term follow-up of 1,347 Australian women aged ≥ 70 years, those with higher vitamin K1 intake performed better in muscle function tests (grip strength and Timed Up and Go test) and had a 16% to 26% lower risk of fall-related hospitalizations. The risk of fall-related hospitalization plateaued at a vitamin K1 intake of approximately 100 μg/d, with associations stabilizing at 70–100 μg/d after multivariate adjustment (15).

Another study of 633 community-dwelling adults aged 55–65 years found that higher vitamin K status was associated with better physical function in women, as assessed by walking tests, chair rise tests, and dressing time (102). Additionally, regardless of sex, stronger grip strength and larger calf circumference correlated with higher vitamin K status (105).

Moreover, a study of 155 hypertensive adults (mean age 62.4 years) revealed that dp-ucMGP was not only associated with increased large artery stiffness, but also showed an inverse correlation with axial muscle mass—elevated dp-ucMGP levels were accompanied by decreased axial muscle mass. Given that dp-ucMGP levels rise in the setting of vitamin K deficiency, these findings suggest that vitamin K may be associated with a favorable relationship with muscle mass in hypertensive populations (38).

Wang et al. (12) analyzed data from the National Health and Nutrition Examination Survey (NHANES, 2011–2018) involving 11,189 participants to explore the relationship between dietary vitamin K intake and skeletal muscle mass. The study found that dietary vitamin K intake was positively correlated with skeletal muscle mass in men (but not in women) and with grip strength within the range of 0–59.871 μg/day. Beyond this threshold, increased vitamin K intake did not yield additional associations in grip strength. Subgroup analysis indicated that among adults aged 18–44 years, muscle mass and strength were significantly associated with dietary vitamin K intake (12).

### 5.2 Randomized controlled trials

In contrast to observational associations, randomized evidence remains limited and heterogeneous. Findings from randomized controlled trials (RCTs) evaluating the effects of vitamin K supplementation on muscle health and physical function remain inconsistent. In a 6-month trial of vitamin K2 (90 μg/day) in 102 patients with type 2 diabetes mellitus (T2DM) aged 50–80 years (HbA1c ≥ 6.5%), supplementation was found to be significantly associated with increases in skeletal muscle mass (SM) and skeletal muscle index (SMI) compared to the placebo group, where these measures decreased (54). Similarly, an 8-week study of vitamin K2 supplementation (90 μg/day) in 84 young women aged 18–40 years with polycystic ovary syndrome (PCOS) showed a notable reduction in waist circumference and body fat mass, along with an association with increased in skeletal muscle mass (109). Conversely, a 12-week trial of vitamin K2 (200 μg/day) in 45 patients with T2DM aged 20–55 years found no significant changes in body composition measures, including muscle mass, fat mass, or fat-free mass (110). Similarly, a 3-year phylloquinone (vitamin K1) supplementation trial conducted in 401 older community-dwelling adults (mean age 69 years) found no significant impact on lean mass (defined as arm lean mass + leg lean mass) or fat mass (103). Additionally, a 6-month menaquinone-7 (MK-7, a form of vitamin K2) supplementation trial in 80 individuals (mean age 77) with a history of vascular disease did not result in associations in grip strength or SPPB scores (111).

Observational studies consistently demonstrate a positive correlation between higher vitamin K levels and better physical function, particularly in older adults. However, evidence from RCTs on the efficacy of vitamin K supplementation remains inconclusive. An analysis of the design and implementation characteristics of existing studies reveals that such trials generally have certain limitations, specifically including small sample sizes, strong heterogeneity of study populations, inconsistent endpoints among different trials, and the lack of muscle-focused primary outcomes in most trials, which is also one of the important reasons for the inconsistency of research evidence. These inconsistencies of these research findings may also stem from variations in vitamin K dosage, form (K1 or K2), population heterogeneity, and study duration. Additionally, differences in participants’ magnesium status may help explain such discrepancies—an issue that is particularly prominent in people with diabetes and older adults, as magnesium deficiency is more common in these groups (112). Furthermore, magnesium itself is involved in protein synthesis as well as muscle contraction and relaxation, suggesting it may play an indirect but significant role in vitamin K’s regulation of muscle health (113, 114). Further research is needed to clarify the specific mechanisms of action of vitamin K, determine its effects across various populations and supplementation regimens, and elucidate the potential interactive role of magnesium. This review encompasses observational clinical studies and randomized controlled trials, as listed in Table 1.

**TABLE 1:** Observational studies and randomized controlled trials (RCTs) evaluating vitamin K and muscle outcomes.

Study (year)	Population (*n*)	Design/duration	Intervention	Main outcomes
Shea et al. (104)	Community-dwelling older adults (*n* = 1,089, mean age 74, 67% female)	Prospective cohort, 4–5 years	Those with ≥ 1.0 nM plasma phylloquinone (the concentration achieved when recommended intakes are met)	Higher plasma phylloquinone associated with better SPPB scores and 20-m gait speed (*p* < 0.05)
van Ballegooijen et al. (102)	Community-dwelling adults (*n* = 633, 55–65 years, 54% female)	Cohort study, 13 years	Mean dp-ucMGP was 376 ± 233 pmol/L	Low vitamin K status associated with lower handgrip strength and poorer functional performance in women
Vidula et al. (38)	Hypertensive adults (*n* = 155, mean age 62.4, 89.6% male)	Cross-sectional	–	Dp-ucMGP levels negatively correlated with axial muscle mass
Sim et al. (15)	Community-dwelling older Australian women (*n* = 1,347, age ≥ 70)	Cohort study, 14.5 years	Vitamin K1 and K2, was 83 ± 31 μg/d and 38 ± 17 μg/d, respectively.	Higher vitamin K1 intake associated with lower odds of slow muscle function (29%) and lower risk of fall-related hospitalization (26%)
Wang et al. (12)	(NHANES, 2011–2018) adults (*n* = 11,189)	Cross-sectional, 8 years	Dietary vitamin K intake	Positive correlation with skeletal muscle mass in men; association with grip strength below ∼60 μg/day
Fulton et al. (111)	Participants with vascular disease (*n* = 80, mean age 77, 44/80 male)	RCT, 6 months	100 μg/day MK-7	No improvement in vascular health or physical function
Shea et al. (103)	Older community-dwelling adults (*n* = 401, mean age 69)	RCT, 3 years	500 μg/day vitamin K1	No significant effects on lean tissue or fat gain
Tarkesh et al. (109)	PCOS women (*n* = 84, 18–40 years)	RCT, 8 weeks	90 μg/day Mk-7	Significant increase in skeletal muscle mass; decrease in waist circumference and fat mass
Karamzad et al. (110)	Adults with anti-T2DM therapy (*n* = 45, 20–55 years)	RCT, 12 weeks	200 μg/day MK-7	No significant differences in fat mass, muscle mass, or bone mass
Zhang et al. (54)	Adults with T2DM (*n* = 102, 50–80 years, HbA1c ≥ 6.5%)	RCT, 6 months	90 μg/day MK-7	Grip strength, skeletal muscle mass (SM), and skeletal muscle mass index (SMI) increased significantly

## 6 Conclusion

In summary, vitamin K is increasingly recognized as a biologically plausible contributor of muscle health through a complex interplay of carboxylation-dependent and independent mechanisms. Its ability to modulate vitamin K-dependent proteins (osteocalcin, MGP, Gas6), suppress inflammation, counter oxidative stress, inhibit ferroptosis, regulate calcium homeostasis, and support mitochondrial function underscores its multifaceted role in preserving muscle mass, strength, and function. Clinical and epidemiological studies generally report that higher vitamin K intake and status correlate with better muscle strength, mass, and mobility, whereas deficiency is associated with frailty, sarcopenia, and physical decline. Notably, observational associations appear most consistent for functional outcomes such as physical performance, grip strength, and falls, while evidence linking vitamin K to muscle mass is more heterogeneous. Although randomized controlled trials (RCTs) have produced variable results, current RCT evidence remains limited and heterogeneous, with inconsistent effects on clinically meaningful muscle outcomes. Differences in study design, vitamin K isoforms, baseline nutritional status, intervention duration, and endpoint selection likely contribute to the mixed findings.

From a translational perspective, optimizing vitamin K status may represent a potentially supportive and adjunctive strategy within broader approaches to healthy aging and muscle preservation. However, routine vitamin K supplementation for the prevention or treatment of sarcopenia is not yet evidence-based, as current interventional data are insufficient to establish consistent clinical benefit. At present, vitamin K should be viewed as a hypothesis-generating nutritional factor rather than a validated therapeutic target. Certain populations, including older adults and individuals with chronic diseases, may be at higher risk of suboptimal vitamin K status; nevertheless, whether targeted supplementation translates into meaningful improvements in sarcopenia-related outcomes remains uncertain. However, from the perspective of supplementation benefits and safety, while vitamin K1 and K_2_ are well-tolerated at standard doses, not only is evidence supporting additional health benefits beyond moderate intake controversial, but relevant long-term safety data are also relatively scarce. Therefore, clinical application should emphasize scientifically controlling supplementation doses based on individual health status and needs, while remaining vigilant against the potential interactions of multi-nutrient combinations. In addition, caution is warranted in individuals receiving vitamin K antagonists, where supplementation may have clinically relevant implications.

Future research should emphasize well-controlled, large-scale clinical trials with uniform endpoints, alongside multi-omics and metabolic profiling, to define the dose–response relationship and isoform-specific actions of vitamin K in human muscle tissue, and also elucidate the potential interactive mechanisms between magnesium and vitamin K. In conclusion, vitamin K represents a promising nutritional target for preserving muscle health and promoting healthy aging. While further validation is required, accumulating mechanistic and clinical evidence suggests that optimizing vitamin K status, particularly in older adults and individuals with chronic diseases, may contribute as a potentially supportive component to strategies aimed at preventing sarcopenia and maintaining functional independence, pending confirmation in adequately powered randomized trials.
